# Supplementary material for: Identification and functional analysis of non-coding regulatory small RNA FenSr3 in Bacillus amyloliquefaciens LPB-18
Source: PeerJ. 2023 May 15;11:e15236. doi: 10.7717/peerj.15236 (PMC10194069; doi:10.7717/peerj.15236)
Supplement: Supplemental Information 4 [file peerj-11-15236-s004.zip › KO/CK-vs-T1_map/map05150.html]

KEGG PATHWAY: Staphylococcus aureus infection - Reference pathway


|  |  |
| --- | --- |
| **Staphylococcus aureus infection - Reference pathway** |  |

[
Pathway menu
| Organism menu
| Pathway entry
| Show description
| User data mapping
]

Staphylococcus aureus can cause multiple forms of infections ranging from superficial skin infections to food poisoning and life-threatening infections. The organism has several ways to divert the effectiveness of the immune system: secreting immune modulating proteins that inhibit complement activation and neutrophil chemotaxis or lysis, modulating the sensitivity to cationic antimicrobial peptides (such as defensin) by increasing the positive net charge of its cytoplasmic membrane, and expression of superantigens that prevent development of a normal immune response or cause an emetic response when ingested.

|  |  |  |
| --- | --- | --- |
| Reference pathway | 184% 150% 122% 100% 82% 67% 55% | 图片下载 |
